# *Helicobacter pylori* Infection among Patients with Dyspepsia and Intrafamilial Transmission

**DOI:** 10.5005/jp-journals-10018-1177

**Published:** 2016-12-01

**Authors:** Mustafa Yalçin, Ayla Yalçin, Göksel Bengi, Selim G Nak

**Affiliations:** 1 Department of Gastroenterology, Dokuz Eylul University Medical Faculty, Ege, Turkey; 2 Department of Gastroenterology, Uludağ University Medical Faculty, Bursa, Turkey.

**Keywords:** Dyspepsia, Eradication, *Helicobacter pylori*, Intrafamilial transmission, Recurrence.

## Abstract

**Introduction:**

Recurrence is an important problem after *Helicobacter pylori* infection, and intrafamilial transmission has an important role in recurrence. In this study, we aimed to investigate the significance of intrafamilial transmission for recurrence development after treatment as well as its usefulness in prevention.

**Materials and methods:**

Of the 109 patients who had dyspepsia and underwent endoscopy, 74 patients had *H. pylori* infection and were enrolled in this study. Infected family members were also detected. Patients were randomly divided into groups I and II, with each group containing 37 individuals. In group I, patients and their infected family members were treated together at the same time. In group II, only the patients were treated. Treatment success was evaluated at the 1st month and evaluation for recurrence was carried out at the 6th month.

**Results:**

*Helicobacter pylori* infection was detected in 67.8% of the patients with dyspepsia. Two patients in each group did not show up at the 1st month control. Eradication was achieved in 63 of the 70 patients (90.0%) who completed their treatment. After 6 months, patients with successful treatment had no recurrence in any of the 32 patients in group I. There were recurrence in 3 of the 31 patients (9.7%) in group II; however, there was no statistically significant difference between the groups (p = 0.113).

**Conclusion:**

Our study showed that eradication treatment in patients and family members with *H. pylori* infection resulted in a decrease in the number of recurrences even though it was not statistically significant.

**How to cite this article:**

Yalçin M, Yalçin A, Bengi G, Nak SG. *Helicobacter pylori* Infection among Patients with Dyspepsia and Intrafamilial Transmission. Euroasian J Hepato-Gastroenterol 2016;6(2):93-96.

## INTRODUCTION

*Helicobacter pylori* is a bacterial pathogen that causes gastroduodenal inflammation leading to gastric and duodenal ulcers and atrophic gastritis.^1^ It is responsible for one of the most common infections worldwide; nearly half of the world’s population harbor the microorganism.^2^ *Helicobacter pylori* infections are generally acquired during childhood via intrafamilial or “mother to child” transmission. *Helicobacter pylori* infection is five times more prevalent in children of infected mothers. *Helicobacter pylori* infections are usually acquired during childhood and more than one strain can colonize in the stomach at this stage. While most of these strains are spontaneously eradicated, one genotype that adapts gastric mucosa and host immune system can permanently colonize in the host.^3^ Intrafamilial transmission, low socioeconomic status, and poor hygienic conditions contribute to the high prevalence of infection.^4,5^

Prevalence and incidence of *H. pylori* infection in gastric mucosa differ between countries, depending on the developmental state and age. While its prevalence varies between 60 and 85% in developing countries, this rate has been reduced down to 10 to 30% in developed countries owing to emphasis on personal hygiene and successful eradication practices.^6^

Recurrence development after the treatment of *H. pylori* is frequent, especially in developing and underdeveloped countries. Annual recurrence rate is 1% in developed countries, whereas this rate reaches up to 30 to 40% in developing countries. Intrafamilial transmission is a known contributor in those high recurrence rates. However, whether infected family members should be treated is still a subject of debate. In this study, we tried to seek answers to these questions.

## MATERIALS AND METHODS

One hundred and nine patients who presented to outpatient of Department of Gastroenterology, Uludağ University Faculty of Medicine with complaints of dyspepsia were informed about the study, and after obtainment of their consent, they were included in the study. Upper gastrointestinal system endoscopy (UGISE) was performed in all patients. *Helicobacter pylori* was detected with rapid urease test [Campylobacter-like organism (CLO) test/Delta Western/Australia]. Patients who were infected with *H. pylori* were enrolled in the study for initiation of eradication treatment. Patients were followed up for 6 months.

Study inclusion criteria were as follows: (1) Patients presenting to outpatient clinic with complaints of dyspepsia, (2) patients who have *H. pylori* infection, (3) patients who are living together with at least three family members for at least 2 years, and (4) adult patients over the age of 18.

Study exclusion criteria were as follows: (1) Patients in whom dyspeptic symptoms are associated with an organic disease other than ulcer and gastritis, such as pancreatic pathologies, gallbladder disease etc., (2) history of previous stomach operation, and (3) pregnancy.

Anamnesis, physical examination, UGISE, rapid urease test during endoscopy, blood tests (hemogram, erythrocyte sedimentation rate, AST, ALT, sodium, potassium, fasting blood glucose, urea, and creatinine), and upper abdominal ultrasonography were performed in all patients. Patients enrolled in the study were randomly divided into groups I and II. In all patients and in family members of patients in group I, the following treatment was administered which represent the most common eradication treatment regime: Lansoprazol 30 mg twice a day, Amoxicillin 1 mg twice a day, and Clarithromycin 500 mg twice a day. The triple therapy was administered for a duration of 14 days.

Whether family members were infected with *H. pylori* was detected with stool antigen test. Premier Platinum HpSA brand stool antigen test was used in the study. The specimens were tested in Uludağ University Microbiology and Infectious Diseases Laboratory.

Study groups were as follows:

*Group I*: Patients and their infected family members living together with the patient were treated together at the same time.

*Group II*: Only the patients were treated. Patients were evaluated for eradication success 1 month after completion of treatment and for development of recurrence 6 months after treatment with stool antigen test. Treated family members were not followed up for recurrence development. Based on these data, it was aimed to seek answers to the following questions: Does intrafamilial transmission has significance in recurring cases of *H. pylori* infection? Is it necessary to treat infected family members of *H. pylori* infected cases? Study plan is shown in Flow Chart 1.

**Flow Chart 1:**
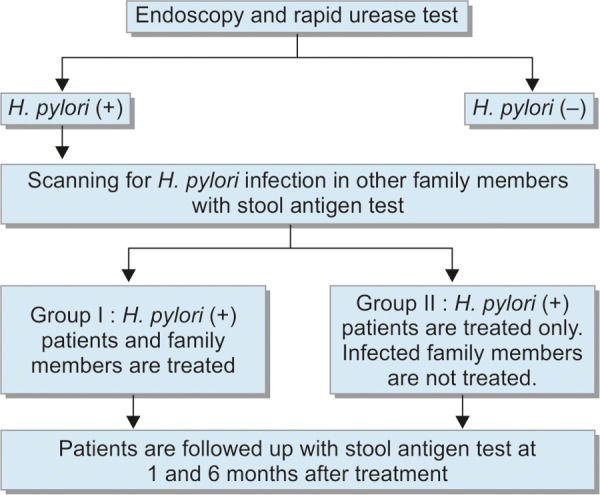
Study plan

### Statistical Analysis

Statistical analyses were carried out using Statistical Package for the Social Sciences (SPSS) 15.0 computer software (SPSS Inc., Software Chicago, IL, USA). Differences between groups were analyzed using Mann–Whitney U test, Pearson’s chi-square test, and Fisher’s exact chi-square test. p-value < 0.05 was accepted as being statistically significant.

## RESULTS

Patients underwent UGISE, and rapid urease test (CLO) was performed. Seventy-four patients (67.8%) were infected with *H. pylori* and enrolled in the study (Table 1). Seventy-four patients (25 men, 49 women) who met the inclusion criteria were randomly divided into two groups.

**Table 1:** **Table 1:** Frequency of *H. pylori* in patients with dyspepsia

		*Number*		*%*	
CLO test (+)		74		67.8	
CLO test (–)		35		32.2	
Total		109		100	

CLO: Campylobacter-like organism

*Group I: Helicobacter pylori* infected family members of the 37 patients in this group were detected with stool antigen test, and they received eradication treatment together with the patients.

*Group II*: Eradication treatment was administered in 37 patients in this group. Family members were not treated.

Demographical properties in each group are presented in Table 2. Mean ages were 40.6 ± 13.76 years in group I and 40.5 ± 9.75 years in group II. There was no statistically significant difference between groups according to Pearson’s chi-square method (p = 0.367). Most of our patients were determined to have moderate to good socioeconomic state. The findings in UGISE are presented in Table 3. Both groups had similar frequencies of stomach ulcers, whereas there were more duodenal ulcers, gastritis, and sliding hernia in group I. There were esophagitis in 10 patients in group I and in 9 patients in group II. Number of family members living together with the patients in groups I and II were determined (Table 4). Totally 125 family members were determined in group I. Twenty-six individuals (26%) were spouses of patients, 99 individuals consisted of children, parents, grandchildren, and daughter-in-law. There were 119 family members in group II, 34 of them (28.5%) were spouses of patients, and 85 individuals consisted of others (Table 4). Stool antigen test results of family members are presented in Table 5. In group I, 34 of the 125 individuals (27.2%) were infected with *H. pylori*; in group II, 34 of the 115 individuals (28.5) were infected. When the spouses were evaluated, 9 individuals in group I and 10 individuals in group II were infected with *H. pylori* (p = 0.614). When all patients were evaluated as a whole, prevalence of *H. pylori* infection was found as 27.8%.

**Table 2:** **Table 2:** Demographical properties of patients

		*Group I*		*Group II*	
Number of cases		37		37	
Age (mean) (years)		40.6 ± 13.76		40.5 ± 9.75	
Female		22		27	
Male		15		10	
Good socioeconomic status		15		17	
Moderate socioeconomic status		19		20	
Poor socioeconomic status		3		0	

**Table 3:** **Table 3:** Findings in upper gastrointestinal system endoscopy

		*Group I*		*Group II*		*Total*	
Stomach ulcer		1		1		2	
Duodenal ulcer		5		3		8	
Gastritis		14		10		24	
Gastroesophageal reflux		10		9		19	
Sliding hiatal hernia		4		2		6	
Normal		5		13		18	
Total		37		37		74	

**Table 4:** **Table 4:** Family members in groups living together with the patient

		*Group I*		*Group II*		*Total*	
Spouse		26		34		60	
Others		99		85		184	
Total		125		119		244

**Table 5:** **Table 5:** Number of family members and stool antigen test results

		*Group I*				*Group II*					
		*H. p (+)*		*H. p (–)*		*H. p (+)*		*H. p (–)*		*Total*	
Spouse		9		17		10		24		60	
Others		25		74		24		61		184	
Total		34		91		34		85		244	

H. p: Helicobacter pylori

Following a treatment for 14 days, patients were called for assessment at 1st month. Two patients in each group did not continue with the study, so the study continued with 35 patients in each group. *H. pylori* eradication was evaluated with stool antigen test. Treatment was unsuccessful in 3 patients in group I (8.57%) and in 4 patients in group II (11.4%). Eradication success was determined as 90% in all patients.

Patients were called for evaluation again at the 6th month. According to evaluation of recurrence with repeat stool antigen test for *H. pylori*, there was no recurrence in group I, and there was recurrence in 3 patients in group II (9.7%). There was no significant difference between the two groups according to Fisher’s exact chi-square test (p = 0.113) (Table 6). The outcome of the study has been summarized in Flow Chart 2.

**Table 6:** **Table 6:** Stool antigen test results of the patients at 1st and 6th months after treatment

		*Group I*				*Group II*					
		*H. p (+)*		*H. p (–)*		*H. p (+)*		*H. p (–)*		*Total*	
1st month		3		32		4		31		70	
6th month		0		32		3		28		63	

H. p: Helicobacter pylori

**Flow Chart 2:**
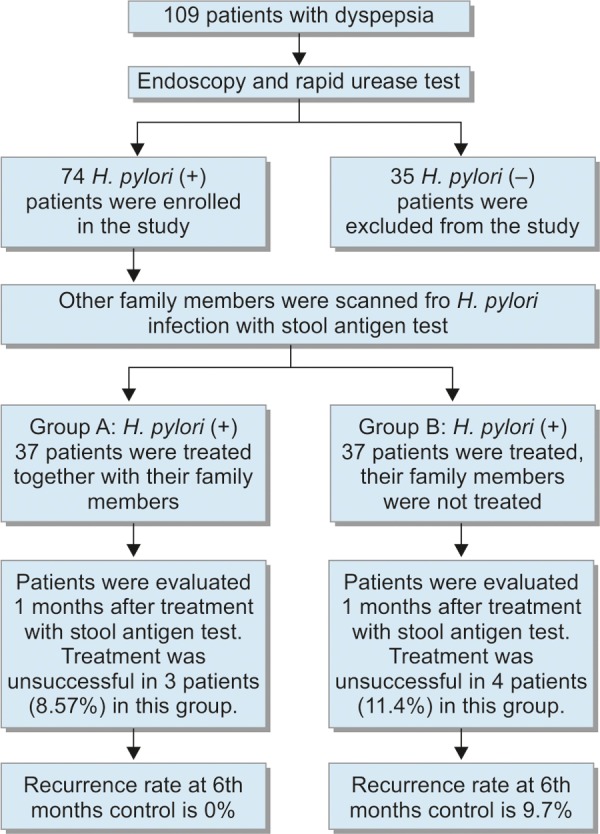
Study plan and findings

## DISCUSSION

More than 50% of the world population is believed to be infected with *H. pylori* today. The prevalence is about 5 to 10% in developed countries, whereas this rate reaches up to 70 to 90%, and even 100% in certain age groups in underdeveloped and developing countries.^3,7,8^ Geographical distribution of the infection is closely associated with socioeconomic status. There are even differences in seroprevalence between different races in developed countries. As an interesting difference, *H. pylori* infection incidence is high in childhood age in developing countries. In developed countries, prevalence of *H. pylori* in childhood age is low, and the prevalence increases with age. The peak level almost never exceeds 50% in these countries.^9^

Turkey has a resemblance to developing countries in terms of *H. pylori* prevalence. However, although, i.e., what the individual studies indicate, general prevalence of *H. pylori* infection in Turkey is not exactly known. Studies investigating *H. pylori* presence in selected patient groups with invasive methods report its prevalence between 41 and 96%.^10^ In our study, we determined *H. pylori* prevalence in patients with dyspepsia in Bursa as 67.8%. This result is consistent with the results of previous epidemiological studies.

*Helicobacter pylori* can be detected in stool, vomit, and sputum, and infection is possibly transmitted via oral to oral, fecal to oral, and gastro to oral routes. Major source of transmission appears to be intrafamilial.^4,5^ Especially mother is the major source of infection at childhood age, while spouses are the major source in adulthood. In areas with high infection rates, *H. pylori* infection appears to be preventable by eradication in family members.^11-13^ Based on these reports, we thought we could reduce recurrences if we treated family members in order to prevent intrafamilial transmission.

We divided 74 patients infected with *H. pylori* randomly into two groups. In both groups, we detected whether family members living together with the patient were infected with *H. pylori*. In group I, we administered eradication treatment to patients together with the infected family members. In group II, we treated the patients only. We evaluated treatment success at the 1st month and recurrence rates at the 6th month using stool antigen test. Eradication success for *H. pylori* was 90% at 1st month in our study. At the 6th month, there was no recurrence in group I, and there were recurrences in 3 patients (9.7%) in group II. There was no statistically significant difference between the two groups according to Fisher’s exact test (p = 0.113). However, administering eradication treatment to family members decreased recurrences.

Our study showed that eradication treatment in patients and family member with *H. pylori* infection resulted in a decrease in the number of recurrences which was not statistically significant. However, since the number of recurrences will increase in the long-term follow-up, we think the treatment of infected family members is necessary.
